# Catalytic Behavior of Iron-Containing Cubic Spinel in the Hydrolysis and Hydrothermolysis of Ammonia Borane

**DOI:** 10.3390/ma14185422

**Published:** 2021-09-19

**Authors:** Oksana V. Komova, Valentina I. Simagina, Alena A. Pochtar, Olga A. Bulavchenko, Arcady V. Ishchenko, Galina V. Odegova, Anna M. Gorlova, Anna M. Ozerova, Inna L. Lipatnikova, Elena S. Tayban, Svetlana A. Mukha, Olga V. Netskina

**Affiliations:** Boreskov Institute of Catalysis SB RAS, Lavrentieva Av. 5, 630090 Novosibirsk, Russia; simagina@catalysis.ru (V.I.S.); po4tar@catalysis.ru (A.A.P.); obulavchenko@catalysis.ru (O.A.B.); arcady.ishchenko@gmail.com (A.V.I.); odegova@catalysis.ru (G.V.O.); gorlova@catalysis.ru (A.M.G.); ozerova@catalysis.ru (A.M.O.); lil@catalysis.ru (I.L.L.); tes@catalysis.ru (E.S.T.); msa@catalysis.ru (S.A.M.); netskina@catalysis.ru (O.V.N.)

**Keywords:** ammonia borane, hydrolysis, hydrothermolysis, ferrite, copper, cobalt, magnetite, copper oxide

## Abstract

The paper presents a comparative study of the activity of magnetite (Fe_3_O_4_) and copper and cobalt ferrites with the structure of a cubic spinel synthesized by combustion of glycine-nitrate precursors in the reactions of ammonia borane (NH_3_BH_3_) hydrolysis and hydrothermolysis. It was shown that the use of copper ferrite in the studied reactions of NH_3_BH_3_ dehydrogenation has the advantages of a high catalytic activity and the absence of an induction period in the H_2_ generation curve due to the activating action of copper on the reduction of iron. Two methods have been proposed to improve catalytic activity of Fe_3_O_4_-based systems: (1) replacement of a portion of Fe^2+^ cations in the spinel by active cations including Cu^2+^ and (2) preparation of highly dispersed multiphase oxide systems, involving oxide of copper.

## 1. Introduction

Ammonia borane (NH_3_BH_3_, AB) is an extensively studied hydride and dozens of review articles have already appeared discussing the results of these studies [1,2,3,4,5,6,7,8,9,10]. The high hydrogen content of this hydride (19.6 wt%) allows it to be applied in a variety of fields: as hydrogen storage systems to generate hydrogen for the needs of hydrogen economy [1,2,3,4,5,6,7,8,9,10], as a component of fuels [11,12], as a reducing agent in fine organic synthesis [13] and in the synthesis of metallic nanoparticles [14,15].

Different ways have been employed to release hydrogen from ammonia borane. Processes of its solid-state dehydrogenation from different hydride-containing materials have been described [5,7]. Processes of its dehydrogenation in organic solvents are also studied [16] (including with the use of catalysts [17]). Catalytic hydrolysis of AB is a simple and most widely used process (1):

NH_3_BH_3_ + 3H_2_O → NH_3_ + H_3_BO_3_ + 3H_2_↑ (1)

NH_3_ +H_2_O ↔ NH_4_^+^ + OH^−^

This process is traditionally carried out in an excess of water at room temperature in the presence of various catalysts [8,9,10,18,19]. Because of the high content of water, this process has a low value of gravimetric hydrogen density (GHD < 1 wt%), which is calculated using the ratio of the mass of released hydrogen to the total mass of all reactants and components of the hydrogen-generating system. 

Hydrothermolysis is another water-employing process for the release of hydrogen from AB [20,21,22,23] where a highly exothermic process of AB hydrolysis (1) is coupled with its thermolysis (solid-state dehydrogenation) (2), (3):

xNH_3_BH_3_ = [NH_2_BH_2_]_x_ + xH_2_↑    ~100 °C     GHD = 6.5 wt% (2)

[NH_2_BH_2_]_x_ = [NHBH]_x_ + xH_2_↑   ~150 °C     GHD = 13 wt% (3)

The process takes place with a high rate of hydrogen generation and with a high yield of hydrogen under the conditions when particles of the hydride wetted with a small quantity of water are heated in a reactor at a temperature of the external heating of >80 °C. The addition of a catalyst facilitates an increase in the rate of hydrogen evolution and a reduce the temperature of the reactor heating [24,25]. Thus, high values of GHD of >7 wt% have been achieved at relatively low temperatures. The effectiveness of the process is rather strongly dependent on the reactor design, the way of reactants loading into the reactor as well as on the conditions of the experiment.

Since AB is a reducer, using ex situ-reduced catalysts is not the only option in the dehydrogenation processes, but a catalytically active state of the catalysts can also be formed in situ using the compounds of active metals added in the reaction medium [26,27,28,29]. It has been found that the in situ-forming catalysts have higher activity [26,27,28]. Let us consider in more detail the use of oxides in the reactions of AB hydrolysis.

An analysis of the literature shows that copper is one of the most important components of the catalysts for AB hydrolysis [10,30]. In 2008, Kalidindi et al. [26] showed the activity of the catalysts decreases in the order Cu_2_O > Cu@Cu_2_O > Cu. It has been confirmed by X-ray diffraction analysis (XRD) that, in the course of the reaction, a reduction of Cu_2_O to Cu^0^ occurs. It was suggested by the authors that the reduction of Cu_2_O in the reaction medium was accelerated by the formation of hydridic Cu-H species. Later, the assumption that the ability of copper to be easily reduced in an aqueous solution of AB to form active Cu-H species was confirmed in a series of studies exploring the activity of copper-containing cobalt oxides: CuCo_2_O_4_ [31,32], Cu_0.6_Ni_0.4_Co_2_O_4_ [33], Co_0.92_Cu_0.08_Co_2_O_4_@Co_0.88_Cu_0.12_Co_2_O_4_ with a «yolk-shell» structure [34], Mo-doped Cu_0.5_Ni_0.5_Co_2_O_4_ [35]. In these studies, it was confirmed by X-ray photoelectron spectroscopy (XPS) that the addition of copper accelerates the reduction of other metal cations comprising the complex oxide. This evidence was explained as follows: Cu^2+^ (0.337 V vs. SHE) are primarily reduced in the reaction medium to form Cu^0^ particles containing hydridic Cu-H bonds on their surface. It is these active hydridic species that promote the reduction of other cations in the oxide. This results in the formation of a multi-component active state of the catalyst immobilized on the rest of the oxide matrix, showing a synergic effect in the AB hydrolysis. Such regularities have also been observed in the case of other complex oxides: Co_0.8_Cu_0.2_MoO_4_ [36] and Co_3_O_4_/CuMnO_4_ [37]. In the latter work, the copper of the CuMnO_4_ accelerated the formation of a cobalt-active component from the separated crystalline phase of Co_3_O_4_ contacting with CuMnO_4_. As a result, the induction period characterizing the long process of reduction of Co_3_O_4_ in the reaction medium disappeared [37]. A similar synergic effect has been observed for the nanosized particles of Co_3_O_4_, whose surface was doped by copper oxide [38] and in the the CuO-NiO/Co_3_O_4_ catalytic system [39].

Unlike the studied cobaltites and molybdates, the application perspectives of Fe_3_O_4_ and ferrrites of transition metals for the AB hydrolysis have not yet been completely investigated. Traditionally, these oxides have been proposed for applications such as magnetically removable supports [40,41,42,43,44]. To prevent the active component from interacting with the support, protecting the support surface using an inert layer of a polymer or silica was proposed. It was also shown that CoFe_2_O_4_ and Fe_3_O_4_ used as supports were themselves not active in this process [43,44].

The addition of copper into these oxides and its effect on the catalytic activity in the water-employing process of AB dehydrogenation remain practically unstudied. This is, in part, explained by the fact that in an article published in 2018 [45]—a series of metal ferrites prepared by precipitation were tested in the process of AB hydrolysis, showing rather low activity which increased in the order NiFe_2_O_4_ < CuFe_2_O_4_ < ZnFe_2_O_4_ < MnFe_2_O_4_, i.e., nickel- and copper-ferrite-containing metals, usually active in the AB hydrolysis—demonstrated low activity. On the other hand, in 2016 the supported CuFe_2_O_4_/rGO (rGO is reduced graphene oxide) has been successfully used in the hydrolysis of NaBH_4_ [46], which is a stronger reducer than AB.

Because of the lack of sufficient information on the ferrites of copper, in 2019, we started a series of further experiments [25], which confirmed the inactivity of Fe_2_O_3_ in the water-employing processes of AB dehydrogenation (hydrolysis (1), hydrothermolysis (1)–(3)); this occurred because Fe^3+^ were hardly reduced by a weak reducing agent such as AB. The activity of Fe_3_O_4_ was higher, but the rate of hydrogen generation was extremely low. However, replacement of an amount of Fe^2+^ by Cu^2+^ allowed a large increase in activity both in the AB hydrolysis and AB hydrothermolysis. In AB hydrothermolysis (90 °C), the catalytic action of copper ferrite was observed both at the stage of hydrolysis and thermolysis. TEM and XRD analyses of the reaction products confirmed the reduction of copper ferrite in the reaction medium to form active nanosized particles Fe^0^ and Cu^0^. It was found that there is a relation between copper ferrite activity and its content of Fe^2+^. It was shown that the combustion of a glycine-nitrate precursor is an effective way to synthesize an active copper ferrite. Since its formation takes place at high temperatures and under conditions of a vigorous gas evolution limiting the access of air to the reaction zone, the structure of the forming cubic spinel has a larger content of Fe^2+^, which decreases when the annealing process begins [25,47].

The present paper is a continuation of these studies. The study of the release of hydrogen from solutions of different hydrides (NH_3_BH_3_, NaBH_4_, and NH_3_BH_3_ + NaBH_4_) in the presence of combustion-synthesized cobalt and copper ferrites will once more emphasize the important role of the reduction rate offered by these oxides in the reaction medium. The synergic catalytic effect of the complex mixed Cu-Fe and Co-Fe oxides and the importance of further investigations of the active iron-containing component will be demonstrated. Comparing the activities of combustion products prepared under different synthesis conditions, and having different phase compositions, will help to reveal the key characteristics of an active catalyst (the content of the cubic spinel and the content of copper in its structure as well as the presence in the sample of active copper-containing impurity).

## 2. Materials and Methods

### 2.1. Methods of Synthesis of the Samples under Study

The reagents and the synthesis of the mixed oxides samples are described in the Supplementary Materials. The samples (CuFe-1, CuFe-2, CuFe-3, CuFe-4, CuFe-5, and CoFe) were prepared by combustion of the glycine-nitrate precursors (Figures S1–S3 in the Supplementary Materials). For these combustion products, the values of specific surface area (S_BET_) were ≤7 m^2^⋅g^−1^, the M/Fe molar ratios (M=Cu,Co) were 0.43 ± 0.01 (the details see Supplementary Materials).

The Fe_3_O_4_ sample (S_BET_ = 15 m^2^⋅g^−1^) was prepared by the traditional procedure of precipitation from FeSO_4_⋅7H_2_O and FeCl_3_⋅6H_2_O with molar ratio of Fe^3+^/Fe^2+^ = 2 as described in [25]. The XRD analysis of dried sample confirmed that the observed diffraction pattern corresponded to the structure of Fe_3_O_4_ [PDF 26-1136], the coherent scattering region (CSR) was 11 nm. The CuO sample (S_BET_ = 49 m^2^⋅g^−1^) was prepared by annealing CuCO_3_⋅Cu(OH)_2_ at 300 °C for 4 h. The observed XRD patterns of the product fully corresponded to those of CuO [PDF 45-937]. The CSR was 10 nm. To prepare the 10 wt% CuO/Fe_3_O_4_ composite, 0.3 g of Fe_3_O_4_ and 0.0927 g of CuCO_3_⋅Cu(OH)_2_ were mixed in a mortar and annealed at 300 °C for 4 h. The S_BET_ and CSR for Co_3_O_4_ reagent (GOST 4467-79, SoyuzKhimProm, Novosibirsk, Russia) used in this study were 16 m^2^/g and 60 nm, consequently.

### 2.2. Methods of Investigations

Attenuated total reflection infrared spectroscopy (ATR FTIR) was performed on an Agilent Cary 600 (Agilent Technologies, Santa Clara, CA, USA) spectrometer equipped with a Gladi ATR (PIKE Technologies, Madison, WI, USA) attachment in the range 240–10,000 cm^−1^ without a pretreatment of the samples.

X-ray diffraction analysis (XRD) was performed on a Bruker D8 Advance diffractometer (Bruker AXS GmbH, Karlsruhe, Germany) in the range of angles 10–80° with a step 2θ = 0.05° and the time of accumulation of 5 s in each point using a Lynxeye linear detector. CuK_α_ radiation (λ = 1.5418 Å) was used. A quantitative phase analysis has been performed by the Rietveld method. The results are presented in Table 1 and Table 2. The average coherent scattering regions were determined using the Scherrer formula from the following reflections: 311 for cubic spinel (Fe_3_O_4_, M_1−x_Fe_2+x_O_4_ M = Cu or Co), 111 for CuO, 111 for Cu, 104 for Fe_2_O_3_, 111 for Cu_2_O, 600 for CuFeO_2_, 311 for Co_3_O_4_, and 111 for CoO. The phases were identified using the following data: CuFe_2_O_4_ [PDF 25-283], CoFe_2_O_4_ [PDF 22-1686], Fe_3_O_4_ [PDF 26-1136], CuO [PDF 45-937], Cu_2_O [PDF 5-667], Cu [PDF 4-836], Fe_2_O_3_ [PDF 33-664], CuFeO_2_ [ICSD 98488], CoO [PDF 42-1300], Co (cub.) [PDF 15-0806], Co_3_O_4_ [PDF 42-1267].

The structure of the samples was studied by high-resolution transmission electron microscopy (HRTEM) using a ThemisZ electron microscope (Thermo Fisher Scientific, Waltham, MA, USA) with an accelerating voltage of 200 kV and a limiting resolution of 0.07 nm. Elemental maps were obtained using energy dispersive spectrometer SuperX Thermo Fisher Scientific. Samples for research were fixed on standard aluminum grid using ultrasonic dispersion in ethanol. The scanning electron microscopy (SEM) images were obtained with a JEOL JSM-6460 LV (Jeol, Akishima, Japan) instrument.

The specific surface area (S_BET_) was determined by desorption of argon using a Sorbi-M instrument (Meta, Novosibirsk, Russia).

The Cu, Co and Fe contents were determined by atomic-emission spectrometry with inductively coupled plasma (ICP-AES) using Optima 4300 DV instrument (Perkin Elmer, Shelton, CT 06484-4794, USA).

The differential dissolution (DD) of the combustion products was performed under a flow dynamic regime using the stoichiograph equipped with inductively coupled plasma atomic emission (ICP AES) spectrometer (Baird, Zoeterwoude, Netherlands) [48]. In this method, identification of the phases is based on their different solubilities when the temperature and acidity of the solvent were changed as the process of dissolution went on in time. A powder of the combustion product (≈ 10 mg) was spread over a disposable support made from a sticky polymeric film and transferred into a flow microreactor for dissolution in a flow of solvent (at a flow rate of 3.6 mL⋅min^−1^). The process was started with an aqueous solution of 0.01 M HCl (Ph = 2), which was gradually replaced by 1.2 M HCl, 3 M HCl and 4 M HF (Figure S4 in the Supplementary Materials). The reactor temperature was gradually raised from 22 to 80 °C. The elemental composition of solvent was determined every 5 s from the spectral lines: 324.7 nm (Cu), 238.2 nm (Fe), and 238.8 nm (Co) with a sensitivity of 10^−3^ μg/mL and an error of determination of ≤5%. The kinetic dissolution curves of the elements were transformed into stoichiograms (molar ratios of the elements) (Figure S5). Mathematical processing of the obtained results allows the content and stoichiometric composition of the mixed oxide to be determined, as well as the contents of impurity phases which were obtained by subtracting the main phase of the mixed oxide from the overall curves of the dissolved elements (Figures S5 and S6) [49]. The stoichiometric formula of the mixed phase without oxygen was determined using the average values for selected segment of the stoichiogram with a standard relative deviation of less than 10% and calculated over several tens of the measured points (Figure S5).

### 2.3. Procedures of NH_3_BH_3_ Dehydrogenation

NH_3_BH_3_ (93%) was prepared and characterized as described in [24]. According to XRD, the average size of the CSR was 70 nm. The particle size of the hydride was within 0.2–0.4 mm. The experimental details of the AB hydrolysis and hydrothermolysis have been described in [23,24,25]. The volume of the released hydrogen was measured by the water displacement method in a gas burette. The obtained values were reduced to the N.T.P. and expressed in mole equivalents of the hydrogen released from one mole of NH_3_BH_3_.

The AB hydrolysis was carried out at 40 and 60 °C under stirring. First, 10 mL of distilled water was added into a glass reactor (V = 52.5 mL) preheated to the required temperature; then, AB (38 mg) and the catalyst powder (11.7 mg) were quickly added in succession. The evolving hydrogen was passed through a condenser to the gas burette (100 mL). To establish the effect of the reducing power of the hydride on the rate of activation of the catalyst in the reaction medium and hydrogen evolution, in similar hydrolysis experiments, NH_3_BH_3_ was replaced by NaBH_4_ (46.5 mg) and by NH_3_BH_3_ (32.4 mg) with a small content of NaBH_4_ (6.8 mg).

In the hydrothermolysis experiments, a mechanical mixture of 46 mg of NH_3_BH_3_ and 5.2 mg of the oxide powder (10 wt%) was loaded into a glass reactor (V = 32 mL) to which distilled water was then added in drops using a micropipette (54 μL) until a molar ratio of H_2_O/AB = 2 was achieved. The sealed reactor was immersed into an oil bath preheated to 90 ± 1 °C. In the course of the experiments, the temperature inside the reaction layer and the volume of the evolving hydrogen were measured. Before going to the gas burette, the evolved hydrogen was passed through a trap filled with a 5% solution of CuSO_4_.

## 3. Results and Discussion

### 3.1. Comparison of the Activities of Cobalt, Copper and Iron Oxides in NH_3_BH_3_ Thermolysis and Hydrothermolysis

**Figure 1:**
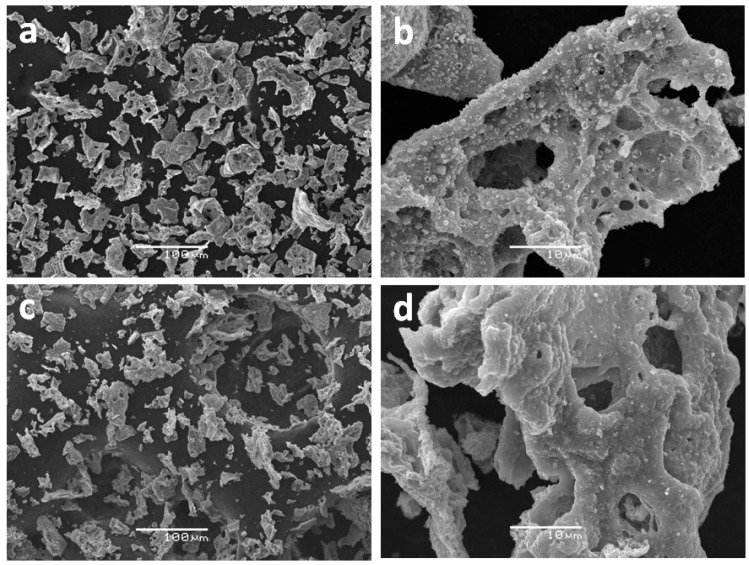
The SEM images of (**a**,**b**) copper ferrite (CuFe-1) and (**c**,**d**) cobalt ferrite (CoFe) prepared by combustion method.

To confirm the promoting action of copper on the reduction of iron in the reaction media of AB hydrolysis and hydrothermolysis, ferrites of copper (CuFe-1) and cobalt (CoFe) have been prepared by combustion of glycine-nitrate precursors (Figures S1 and S3 in the Supplementary Materials) and characterized by ATR FTIR, XRD, DD and SEM. The micrographs (Figure 1) of the obtained combustion products indicated that they have close sizes of their particles and for the most part consist of micro-porous particles with a size smaller than 100 μm. The S_BET_ for CuFe-1 and CoFe was also the same (4 m^2^/g).

In Table 1, the results of the XRD phase analysis are compared with those obtained by DD. Note that, when using XRD, it is more difficult to distinguish between the cubic spinel of Fe_3_O_4_ and those of Cu_1−x_Fe_2+x_O_4_ and Co_1-x_Fe_2+x_O_4_ because of the close parameters of their crystalline structures. The advantages of the combined use of XRD and DD have been demonstrated earlier [25], where DD was used to determine the stoichiometry and content of the main phase of the cubic spinel. The contents of the impurity phases were calculated, as were the rest of the remaining substances, after subtraction of the main phase from the overall dissolution curves of the determined elements.

Using the results obtained by XRD (Table 1) and DD (Figures S5–S7), it is demonstrated that the main phase of the combustion products is a spinel with a cubic structure (Cu_0.67_Fe_2.33_O_4_ or Co_0.90_Fe_2.10_O_4_). Apart from the main phase of the cubic spinel, these methods also reveal the presence of other phases of copper, cobalt and iron. Cobalt ferrite has smaller contents of such impurity phases and, hence, more cobalt is found in the structure of the spinel. We believe this to be associated with the fact that, under the conditions of the vigorous combustion and a limited access of air to the reaction zone, Co^2+^ is less capable of being reduced, which allows it to enter the spinel lattice.

**Figure 2:**
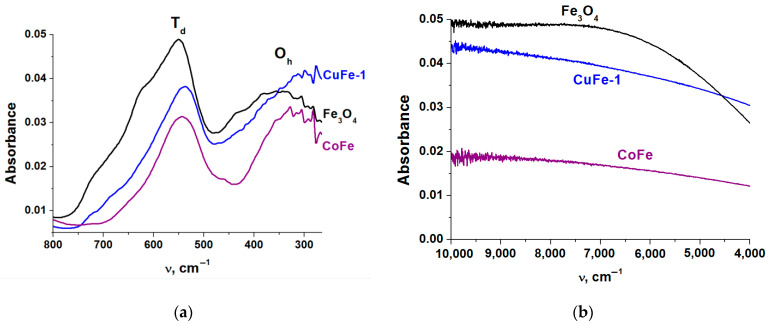
The spectra of Fe_3_O_4_, copper ferrite (CuFe-1) and cobalt ferrite (CoFe) in the region of vibrations of metal–oxygen bonds (**a**) and in the near-infrared region (**b**).

The ATR FTIR spectra in the region of metal-oxygen vibrations are shown in Figure 2a. As in the spectrum of Fe_3_O_4_, the spectra of CuFe-1 and CoFe clearly reveal the presence of two intense absorption bands at 700–500 cm^−1^ and 470–260 cm^−1^, which were to the vibrations of iron in the tetrahedral [FeO_4_] and octahedral [FeO_6_] environments, respectively [50,51]. In the case of ferrites, the shifts in the centers of gravity of these bands are associated with the influence of the copper or cobalt cations on the vibrations of iron, as well as with the possible reduction of the spinel cations leading to the change of the Fe-O bonds [52]. These changes are most characteristic of the vibrations of [FeO_6_]. A more detailed analysis of Fe-containing spinel spectra was given in our previous paper [25].

The higher content of cobalt in the cobalt ferrite as compared with the content of copper in the copper ferrite suggests that there will be a low content of Fe^2+^ in the structure of the cobalt-containing spinel. This suggestion is confirmed by the ATR FTIR spectra in the near-IR region (Figure 2b). It is known that the absorption in this region characterizes the electronic conductivity in Fe_3_O_4_ due to the constant exchange of electrons between Fe^2+^ and Fe^3+^ in the closely lying octahedral positions [53]. The lower level of absorption in the case of CoFe sample confirms the greater extent of the Fe^2+^ replacement by Co^2+^ in this spinel.

**Figure 3:**
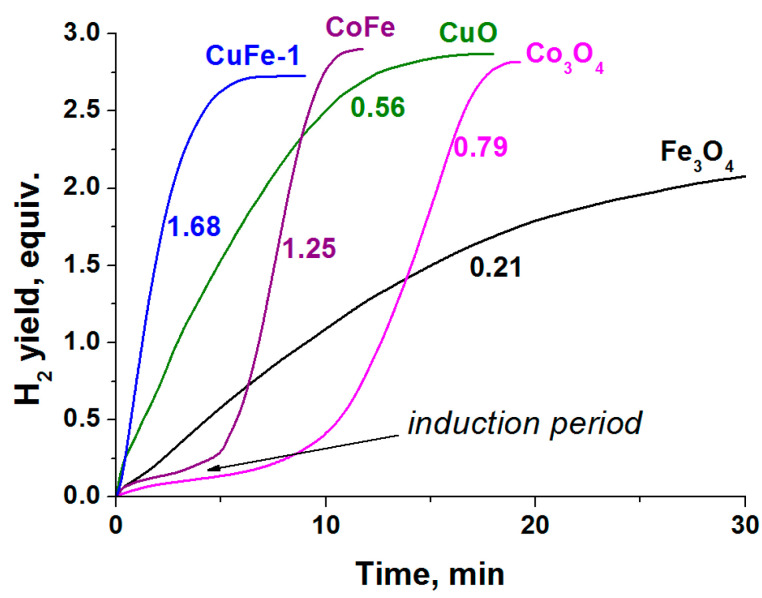
The influence of oxides on the hydrogen generation during catalytic hydrolysis of NH_3_BH_3_ (0.123 M) at 60 °C. The numbers indicate the attainable rate of H_2_ generation expressed as L_H2_⋅min^−1^⋅g^−1^_cat_.

As seen in the results shown in Figure 3, in the case of copper and cobalt ferrites, the rates of hydrogen generation are higher than in the case of individual oxides. Note that CuO, Co_3_O_4_, and Fe_3_O_4_ have higher specific surface areas than CuFe-1 and CoFe. On the contrary, this should improve their interaction with the reaction medium. Considering the low activity of Fe_3_O_4_, higher rates of hydrogen generation in the presence of ferrites are achieved at lower contents of copper or cobalt in the reaction medium than in the case of the oxides of these metals. This indicates a synergic action of the metals comprising the spinel. It should also be noted that the curves of hydrogen generation in the presence of the studied compounds of cobalt show the presence of an induction period (Figure 3). Earlier, when studying the Co_3_O_4_-catalyzed hydrolysis of NaBH_4_ and NH_3_BH_3_, the presence of the induction period has been explained by the slow reduction of cobalt oxide in the reaction medium to form active nanosize cobalt borides [29]. The absence of an induction period in the case of the copper-containing oxides indicates a faster activation of the catalyst in the reaction medium.

**Figure 4:**
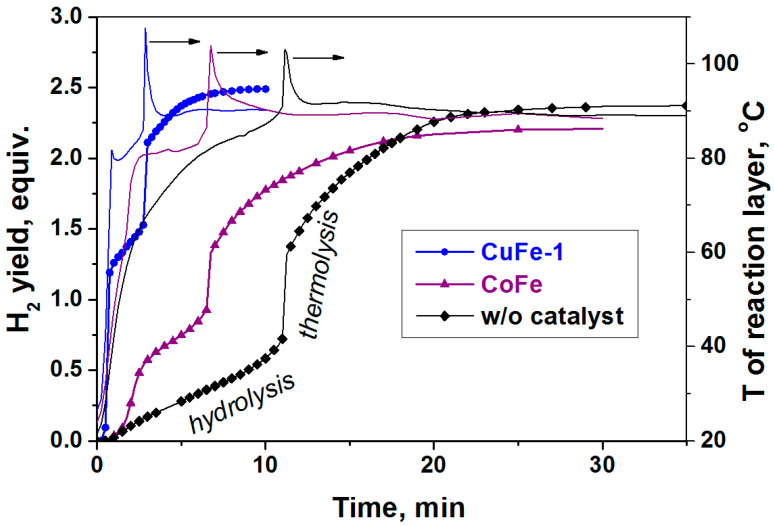
The influence of ferrites on hydrogen generation (symbol + line) and temperature of reaction layer (line) during the catalytic hydrothermolysis of NH_3_BH_3_ at external heating of reactor at 90 °C and a molar ratio of H_2_O/NH_3_BH_3_ = 2.

The activation behavior of the ferrites of copper and cobalt in the process of AB hydrolysis (Figure 3) repeats itself in the stage of hydrolysis in another process—AB hydrothermolysis (Figure 4). We believe that the shorter induction period in the case of CoFe is associated with the higher temperatures of the reactor heating used in this process and the higher concentration of the hydride in the reaction medium. As expected, this confirms the earlier-established correlation between the rate of the AB hydrolysis stage and the time during which the process proceeds to the next stage of the AB thermolysis [23,24,25]. The catalytic systems which were more active in the AB hydrolysis are characterized by faster rates of hydrogen generation and higher yields of hydrogen in the first exothermic stage of AB hydrolysis, which provides a faster transition from the stage of hydrolysis to the stage of thermolysis (Figure 4).

**Figure 5:**
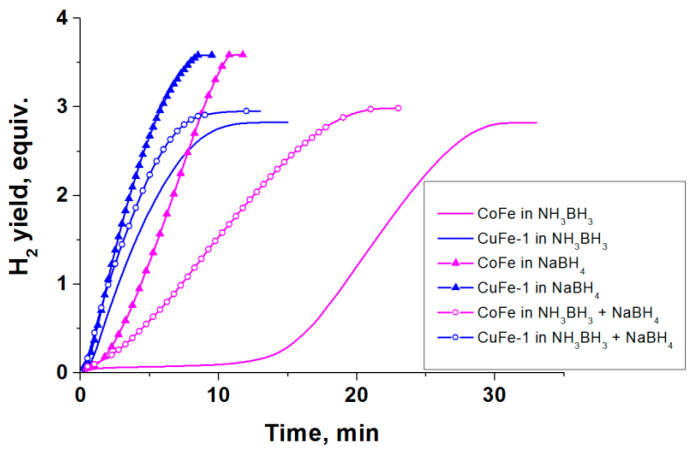
The effect of reductive ability of hydride medium on the hydrogen generation during catalytic hydrolysis of solutions of NH_3_BH_3_ (0.123 M), NaBH_4_ (0.123 M) and NH_3_BH_3_ (0.105 M) with NaBH_4_ (0.018 M) over copper and cobalt ferrites at 40 °C.

The important role of the stage of oxide reduction has been demonstrated in experiments, where CuFe-1 and CoFe samples have been tested in the hydrolysis of NH_3_BH_3_, NaBH_4_ and NH_3_BH_3_ with a small content of NaBH_4_ (Figure 5). These experiments were carried out at a lower temperature (40 °C) in order to lengthen the time of the induction period.

Ammonia borane is known to be a considerably weaker reducer than sodium borohydride [14,29]. The addition of ammonia borane to a small amount of NaBH_4_ enhances the formation of the active component in the reaction medium [29,54] It is seen (Figure 5) that the most remarkable changes were in the case of cobalt ferrite where the stronger reducing power of the reaction medium resulted in an almost ten times shorter induction period in the hydrogen generation curve. The highest rate of hydrogen generation was in the case of the solution of NaBH_4_. In moving from NH_3_BH_3_ to NH_3_BH_3_ + NaBH_4_ and NaBH_4_ solutions, the time of the reaction in the presence of CoFe was reduced from 32 min to 23 min and 11 min, respectively. The changes in the case of CuFe-1 were less pronounced (Figure 5). The time of the reaction shortened from 13 min (NH_3_BH_3_) to 12 min (NH_3_BH_3_ + NaBH_4_) and 8.5 min (NaBH_4_). Overall, these results show that the product of combustion consisting predominantly of the copper-doped cubic spinel Cu_0.67_Fe_2.33_O_4_ shows considerable advantages over the Co_0.90_Fe_2.10_O_4_. Based on our results and the results reported in the literature such a behavior may, in the first place, be explained by the ability of copper ferrite to reduce in the reaction medium to form a catalytically active phase consisting of nanosized Fe^0^ and Cu^0^. The results of the above-made comparison of the activities of cobalt and copper ferrites once more confirm the assumption reported in the literature that the reduction of iron in the oxide structure is accelerated by the forming Cu-H species which have a high hydrogenation power [10]. It allows us to suggest that the formation of the hydridic Cu-H species is of importance not only during the stage of catalyst activation but also in the subsequent stage of the active generation of hydrogen because they slow down the oxidation of the nanosized active component in the aqueous reaction medium.

In the next section, the activity in the processes of AB hydrolysis and hydrothermolysis will be compared for combustion products which were synthesized under different conditions. As a result, the formed combustion products differed in the dispersity, the content of the cubic spinel, the content of Cu in the spinel structure and the content of the impurities. Analysis of these results allowed us to obtain more detailed characteristics of the active catalytic additive.

### 3.2. Effect of the Phase Composition of Products of Combustion of Copper-Iron-Glycine-Nitrate Precursors on Their Activity in NH_3_BH3 Hydrolysis and Hydrothermolysis

As can be seen from Table 2, the prepared products of combustion of copper-iron-glycine-nitrate precursors differ in the content of the cubic spinel, CSR and the content and composition of impurities. For all samples, the Cu/Fe molar ratios determined by ICP-AES were close 0.43 ± 0.01.

**Figure 6:**
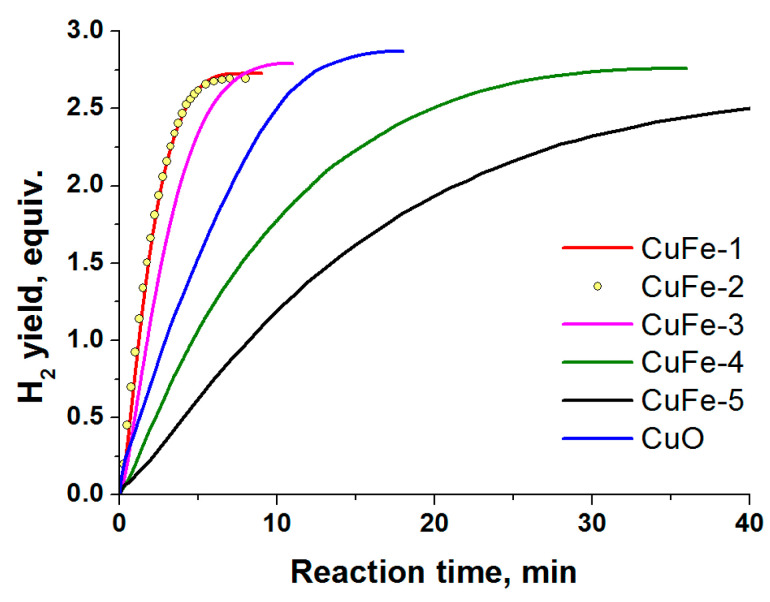
The activity of CuFe-combustion products (Table 2) in the catalytic hydrolysis of NH_3_BH_3_ (0.123 M) at 60 °C.

**Figure 7:**
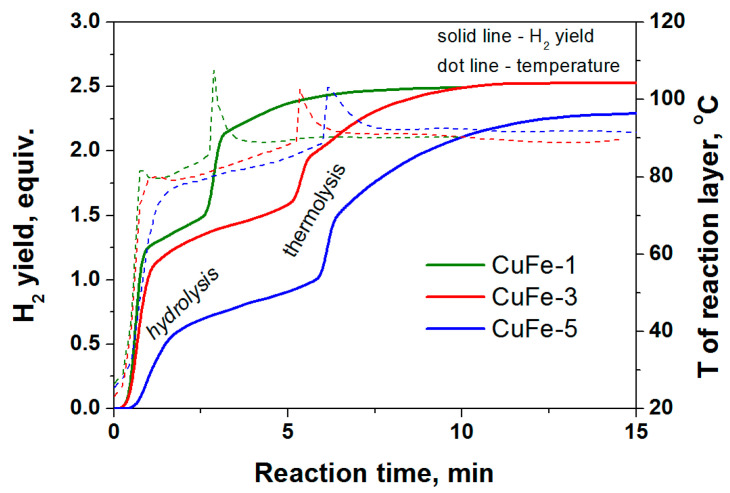
The activity of CuFe-combustion products (Table 2) in the catalytic hydrothermolysis of NH_3_BH_3_ at external heating of reactor at 90 °C and molar ratio of H_2_O/NH_3_BH_3_ = 2. The solid lines are the hydrogen generation curves; the dotted lines are curves of temperature of reaction medium.

The results presented in Figure 6, Figure 7 show that the method of preparation and the characteristics of the combustion products determine their activity in the AB hydrolysis and hydrothermolysis. As expected, the behavior in the AB hydrolysis (Figure 6) repeats itself in the case of AB hydrothermolysis (Figure 7). To find out the most significant parameters of the combustion products, consisting not only of the phase of the cubic spinel but also contain other phases (Table 2), let us first consider the correlation between the product’s activity and its characteristics for two of the studied combustion products: the most active sample, CuFe-1, and the least active sample, CuFe-5.

Let us start with the size of the particles. The SEM analysis (Figure S9) has shown the particles of the little active CuFe-5 sample were the most sintered ones. This is also confirmed by the highest value of CSR for the phase of the cubic spinel (Table 2). Apart from its small dispersity, the least active sample CuFe-5 shows the lowest content of the cubic spinel (34%), the highest content of Fe_2_O_3_ (40%) and a high content of copper in the form of individual phases (Cu^0^ + Cu_2_O). As we showed earlier [47], the formation the mixed copper-iron oxide phase in the case of this sample is complicated not only by the short time available for the components to interact in the reaction zone but also by the high temperatures in the reaction zone and the reduction of copper under the conditions of the vigorous gas evolution which decreases the content of oxygen in the reaction zone. This causes the copper to leave the structure of the spinel [55,56].

**Figure 8:**
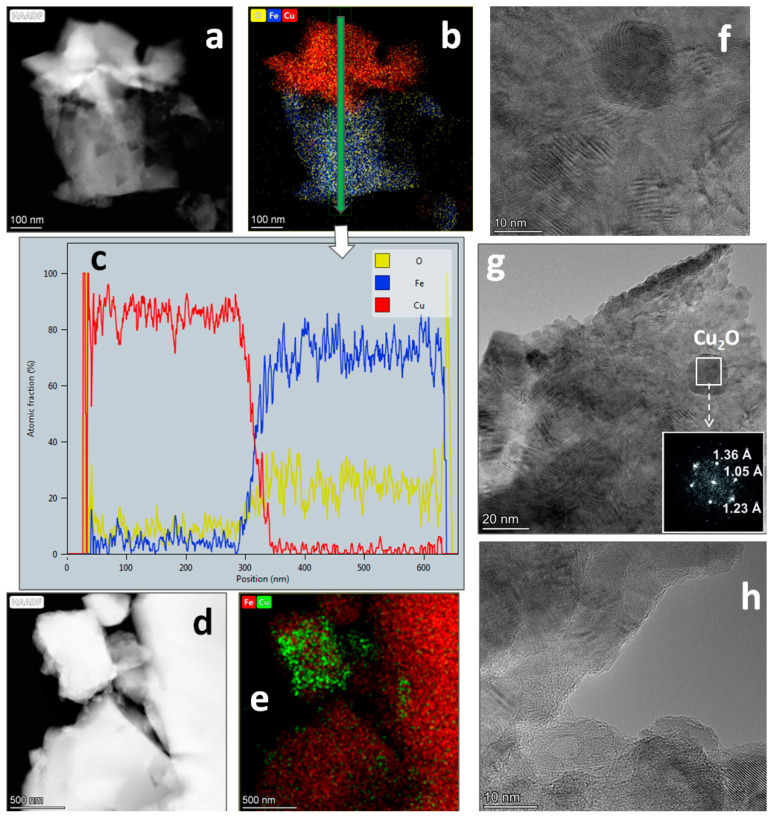
For an inactive CuFe-5 sample before catalytic experiments, (**a**,**d**) dark-field images with (**b**,**e**) the corresponding elemental mappings, (**c**) concentrations distribution at a particle scanning shown at (**b**), (**f**–**h**) HRTEM images, and (**g**) the selected area and its diffraction pattern.

**Figure 9:**
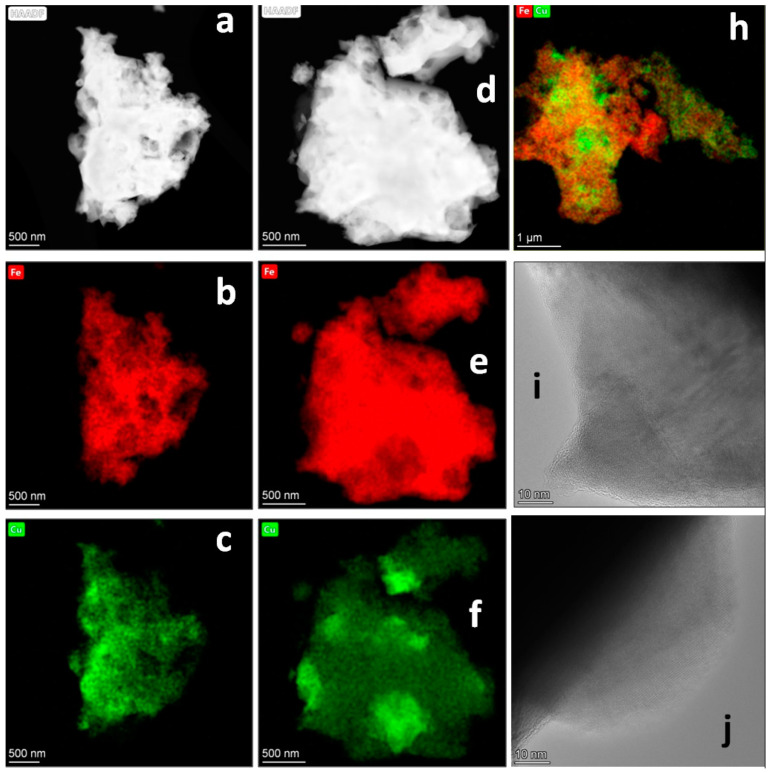
For an active CuFe-1 sample before catalytic experiments, (**a**,**d**) dark-field images with (**b**–**f**) the corresponding elemental mappings, (**h**) elemental mappings for another particle, and (**i**,**j**) HRTEM images.

The phase of the cubic spinel found by XRD is attributable both to the small amount of active Fe_3_O_4_ and the active copper ferrite Cu_1−x_Fe_2+x_O_4_ [25]. In the compared samples, HRTEM and DD were used to assess the presence of the mixed copper-iron oxide phase and determine the spinel content and its stoichiometry. Figure 8 and Figure 9 show the results of the HRTEM analysis for the little-active CuFe-5 and the active CuFe-1.

As shown in Figure 8, the particles of the little-active CuFe-5 mainly consist of individual phases of iron and copper. Nanosized spherical particles also occur (Figure 8f,g) and are attributed to Cu_2_O (PDF 5-667). Regions of mixed oxides containing both copper and iron are rare. The concentration of copper in the particles of iron oxide is generally low. It can, therefore, be suggested that, in the CuFe-5 sample, the cubic spinel (34%) is mainly Fe_3_O_4_ or a slightly Cu-doped Fe_3_O_4_. Additionally, the high-resolution micrographs of this sample indicate the presence of large number of moiré patterns (Figure 8g), which is a consequence of the superposition of periodic layered structures with either slightly different lattice constants or different orientation. Amorphous regions also occur (Figure 8h).

The TEM results for the active CuFe-1 are quite different (Figure 9). All particles of this sample contain both copper and iron, which confirms that the main phase of the cubic spinel (84%) is a mixed copper-iron oxide. It is seen that the stoichiometry Cu/Fe within a single particle is not constant (Figure 9d–h). Regions with a higher and a lower content of copper (or iron) occur. We believe that these regions are the sites of localization of individual phases of copper and iron and of the CuFeO_2_ phase which has a higher Cu/Fe ratio than CuFe_2_O_4_. In the HRTEM images, the moiré patterns are practically not observed (Figure 9i,j), which appears to indicate a greater phase homogeneity of this sample. This is consistent with the XRD data (Table 2).

Thus, the comparison of the two products of combustion (CuFe-1 and CuFe-5) allows us to conclude that the active CuFe-1 product predominantly consist of more finely dispersed particles of copper ferrite with average stoichiometry of Cu_0.67_Fe_2.33_O_4_ and the presence of a number of other phases of copper and iron as an impurity. To answer the question, ‘how important are the contents of the cubic spinel, the copper content in the spinel structure and the presence of other compounds of copper and iron for the catalytic activity?’, the results of XRD analysis obtained for a number of combustion products prepared under different synthesis conditions (Table 2) have been correlated with their activity in the AB hydrolysis (Figure 6).

The following series were obtained:

- The rate of H_2_ generation in the presence of CuFe combustion products (Table 2) in the catalytic AB hydrolysis decreases in the order (Figure 6):
  CuFe-1 ≈ CuFe-2 > CuFe-3 > CuFe-4>CuFe-5;(4)
- The content of the spinel phase in wt% decreases in the order:
  CuFe-1 (84%) > CuFe-2 (73%) > CuFe-3 (66%) > CuFe-4 (48%) > CuFe-5 (34%);(5)
- CSR of the spinel phase in nm increases in the order:
  CuFe-2 (49 nm) < CuFe-3 (52 nm) < CuFe-1 (62 nm) ≈ CuFe-4 (62 nm) < CuFe-5 (77 nm);(6)
- The content of the oxide phases of copper (CuO+Cu_2_O+CuFeO_2_) expressed in wt% of Cu decreases in the order:
  CuFe-2 (9.6%) > CuFe-1 (7.9%) ≈ CuFe-4 (7.4%) ≈ CuFe-3 (7.3%) > CuFe-5 (6.2%);(7)
- The content of the CuO phase in wt% decreases in the order:
  CuFe-2 (12%) > CuFe-3 (6%) > CuFe-1 (3%) > CuFe-4 ≈ CuFe-5 (0%);(8)
- The content of the Cu_2_O phase in wt% increases in the order:
  CuFe-1 ≈ CuFe-2 ≈ CuFe-3 (0%) < CuFe-4 (6%) ≈ CuFe-5 (7%);(9)
- The content of the CuFeO_2_ in wt% decreases in the order:
  CuFe-1 (13%) > CuFe-3 (6%) ≈ CuFe-4 (5%) > CuFe-2 ≈ CuFe-5 (0%);(10)
- The content of the Cu^0^ phase (CSR > 150 nm) in wt% increases in the order:
  CuFe-1 (0%) < CuFe-2 (6%) < CuFe-3 (14%) ≈ CuFe-4 (15%) < CuFe-5 (18%);(11)
- The content of all impurity phases of copper (all of the oxide phases of copper + Cu^0^) expressed in wt% of Cu increases in the order:
  CuFe-1 (7.9%) < CuFe-2 (15.6%) < CuFe-3 (21.3%) ≈ CuFe-4 (22.4%) < CuFe-5 (24.2%);(12)
- The content of the Fe_2_O_3_ phase in wt% increases in the order:
  CuFe-1 (0%) < CuFe-3 (8%) ≈ CuFe-2 (9%) < CuFe-4 (26%) < CuFe-5 (40%).(13)

The comparison of these results allows us to obtain more detailed information concerning the composition of the active catalyst. Note that activity is not explained by the dispersity (CSR, S_BET_) of these samples. It was shown that the activity of the studied combustion products (4) correlates with their content of the cubic spinel (5). The higher the content of the cubic spinel, the higher the activity. The activity of the studied combustion products tends to decrease with the increasing fraction of the impurity phases of copper (CuO + Cu_2_O + CuFeO_2_ + Cu^0^) (12) and the increasing content of the inactive phase Fe_2_O_3_ (13). This explains the high activity of CuFe-1, which mainly consists of the cubic spinel (83%) with a relatively large extent of the Fe^2+^ substitution by Cu^2+^ (Cu/Fe = 0.29 in moles).

On the other hand, from the results of the present study, it follows that the high content of copper in the lattice of the cubic spinel is not an indispensable condition for a high catalytic activity. In contrast to CuFe-1, the spinels of the other products are characterized by rather low theoretical molar ratios Cu/Fe < 0.1, since a considerable portion of copper in these samples is present in the form of individual compounds (Table 2). This is especially characteristic of CuFe-2 sample, whose activity in the AB hydrolysis (Figure 6) is comparable to that of CuFe-1 at an XRD derived content of the cubic spinel of 73%. A DD analysis of this sample (Table 3) confirmed the XRD results and allowed us to obtain more exact values of the Cu/Fe molar ratio in the spinel of this sample. It was found to be 0.06 which corresponds to the Cu_0.16_Fe_2.84_O_4_ composition.

Further correlation between the order of activities of CuFe-samples (4) and their contents of CuO (8), Cu_2_O (9), CuFeO_2_ (10), and Cu^0^ (11) phases allowed us to make the following conclusions. The active samples CuFe-1, CuFe-2 and CuFe-3 do not contain Cu_2_O. The high activity cannot also be related to the presence of coarsely dispersed metallic copper. On the contrary, it is the low-active samples CuFe-4 and CuFe-5 that contain a considerable amount of reduced copper (15–18 wt%) (11), a high content of Fe_2_O_3_ (13), a low content of the spinel (5) and a Cu_2_O impurity (9). According to XRD, the value of CSR of Cu^0^ formed upon the combustion is >150 nm (Table 2), which substantially exceeds the CSR of the active copper forming from the oxides in the reaction medium of ammonia borane (≤40 nm) [25]; this seems to be responsible for the low activity of such samples. There is no correlation between the activity and the presence of the CuFeO_2_ phase. For example, this phase is not present in the active CuFe-2 but is found in the low-activity CuFe-4 and in the active CuFe-1.

In our opinion, the high activity of CuFe-2 comparable to that of CuFe-1 may be related to its high content of CuO (12%) (Table 2, (8)). The CuO phase is also found in the most active sample CuFe-1 (3%) and in the sample CuFe-3 (6%)—the next member in the order of activities. The least active samples CuFe-4 and CuFe-5 do not contain CuO.

This allowed us to suggest that the active combustion product may not only show a relatively high extent of Fe^2+^ substitution by Cu^2+^ in spinel structure but also it may predominantly consist of Fe_3_O_4_ (including with the presence in its structure of a small content of copper) together with an amount of the active CuO phase. We believe that as in the case of mixed Cu-Fe oxide phase, the reduction of CuO at the CuO–Fe_3_O_4_ interface to form active Cu-H sites must lead to the reduction of iron in the structure of Fe_3_O_4_. A similar effect has been described for the systems CuO/Co_3_O_4_ [38] and CuO-NiO/Co_3_O_4_ [39], which are being investigated for use in AB hydrolysis. No such evidence has been reported for Fe_3_O_4_. It should be noted that the potential of active iron-containing components in the catalytic processes discussed in this study is still little studied. However, there have been some reported results dealing with the promoting action of iron on active nickel and cobalt-active components [8,10,57,58,59,60].

**Figure 10:**
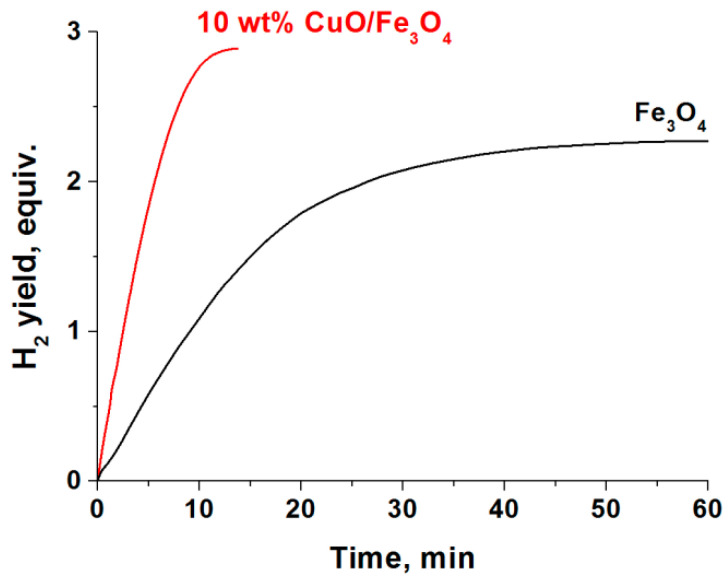
The change of activity of Fe_3_O_4_ in the catalytic hydrolysis of NH_3_BH_3_ (0.123 M) at 60 °C upon its modification by CuO (10 wt%).

To verify this assumption, in an experiment an amount of Fe_3_O_4_ was ground with a basic carbonate of copper and the mixture was calcined in air at 300 °C for 4 h to produce a 10 wt% CuO/Fe_3_O_4_. We were aware that this method of preparation is not the most optimal and efficient, since a mere calcination of such a mixture cannot ensure the formation of the active nano-dispersed states of CuO and, in addition, there a partial oxidation of Fe_3_O_4_ to γ-Fe_2_O_3_ will take place; the latter is less active than Fe_3_O_4_ [25]. However, it was a fast way to verify our assumptions. Indeed, the data in Figure 10 show that Fe_3_O_4_ modification by a small amount of CuO facilitates a considerable increase in its activity. The observed activity of 10 wt% CuO/Fe_3_O_4_ exceeds that of the analogously prepared bulk CuO. These results may help to find new approaches to the creation of novel Fe_3_O_4_-based active materials for water-employed AB dehydrogenation.

## 4. Conclusions

The catalytic activity of Fe_3_O_4_ in the reactions of ammonia borane hydrolysis and hydrothermolysis has been compared with the activity of copper and cobalt ferrites with the structure of a cubic spinel prepared by combustion of glycine-nitrate precursors. In the process of AB hydrothermolysis, a highly exothermic reaction of AB hydrolysis is coupled with the subsequent stage of AB thermolysis. Note that, with this process, high values of GHD (>7 wt%) can be achieved at relatively low temperatures of the external heating (>80 °C) [20,21,23,24,25].

The rate of hydrogen generation was found to change in the order 

Cu_0.67_Fe_2.33_O_4_ > Co_0.90_Fe_2.10_O_4_ > Co_3_O_4_ > CuO > Fe_3_O_4_. This order was not explained by the dispersity of studied samples, but depended on their chemical composition. The obtained results indicated a synergic action of the metals comprising the spinel. The advantages of using copper ferrite are not only its high activity in the AB hydrolysis and hydrothermolysis, but also the absence of an induction period in the curves of hydrogen generation which is associated with its fast reduction in the reaction medium to form active nanodispersed Fe^0^ and Cu^0^ [25]. A comparison of the results for the activity of copper and cobalt ferrites in reaction media having different reducing powers (solutions of NH_3_BH_3_, NH_3_BH_3_+NaBH_4_ and NaBH_4_) confirmed the easier reduction of the copper-containing oxide. It has been suggested [10] that this happens because of the easier reduction of copper in the hydride medium to produce hydridic Cu-H species accelerating reduction of other metals in the oxide structure. The activating action of the compounds of copper, including its oxides, is well known and has been employed in the processes of hydrogenation of different organic substrates where ammonia borane is used as a mild reducer [61,62,63].

The obtained results allow us to suggest that the formation of the active hydridic Cu-H species is of importance not only for the initial stage of activation, but also for the later stage of the active generation of hydrogen, preventing the oxidation of the active, nanosized component in the aqueous medium.

To reveal the most significant parameters of the active catalytic system on the basis of copper-containing spinel, the activity of a series of combustion products which differed in the their dispersity has been analyzed: the contents of the cubic spinel, the content of copper in spinel structure and the content and composition of impurity phases. Based on the obtained results, it was suggested that the catalyst may be active not only when a portion of Fe^2+^ in cubic spinel structure is replaced by Cu^2+^, but also when it consists of Fe_3_O_4_ with an amount of CuO.

Thus, the obtained results show the high potential of the copper-modified Fe_3_O_4_ as a precursor, allowing the active catalytic states to be formed directly in the reaction medium. We believe that such catalytic systems may be improved further by replacing a portion of Fe^2+^ cations in the spinel structure by active cations (Ni^2+^, Co^2+^, etc.) together with Cu^2+^ or by preparation of finely dispersed multi-phase oxide systems, involving oxide of copper.

## Figures and Tables

**Table 1:** The comparison the results of X-ray diffraction (XRD) method and differential dissolution (DD) for the samples of copper and cobalt ferrites prepared by combustion synthesis.

Sample	XRD (wt%)	DD (wt%) ^1^
CuFe-1	84% Cu_1−x_Fe_2+x_O_4_ (cub. spinel) CSR = 62 nm 13 % CuFeO_2_ CSR = 130 nm 3% CuO	75.9% Cu_0.29_Fe_1_ ^2^ 14.4% Cu_1_ 9.7% Fe_1_
CoFe	90% Co_1−x_Fe_2+x_O_4_ (cub. spinel) CSR = 93 nm 8% CoO CSR = 40 nm 3% Co (cub.)	87.4 % Co_0.43_Fe_1_ ^3^ 8.2% Co_1_ 4.4% Fe_1_

^1^ Since oxygen is not determined by the DD method, the stoichiometric formulas of the corresponding phases are traditionally presented without oxygen. ^2^ Corresponds to Cu_0.67_Fe_2.33_O_4_ formula. ^3^ Corresponds to Co_0.90_Fe_2.10_O_4_ formula.

**Table 2:** The results of X-ray diffraction method and specific surface values for the different combustion products obtained in different regimes.

N°	Sample	S_BET_ (m^2^/g)	Phase Composition (wt%)
1	CuFe-1	4	84% spinel (cub.) CSR = 62 nm 3% CuO 13% CuFeO_2_ CSR = 130 nm
2	CuFe-2	7	73% spinel (cub.) CSR = 49 nm 6% Cu CSR > 150 nm 12% CuO CSR = 20 nm 9% Fe_2_O_3_ CSR = 31 nm
3	CuFe-3	7	66% spinel (cub.) CSR = 52 nm 14% Cu CSR > 150 nm 6% CuO 8% Fe_2_O_3_ CSR = 54 nm 6% CuFeO_2_
4	CuFe-4	3	48% spinel (cub.) CSR = 62 nm 15% Cu SCR > 150 nm 6% Cu_2_O 26% Fe_2_O_3_ CSR = 93 nm 5% CuFeO_2_
5	CuFe-5	<1	34% spinel (cub.) CSR = 77 nm 18% Cu CSR > 150 nm 7% Cu_2_O CSR = 28 nm 40% Fe_2_O_3_ SCR = 71 nm

**Table 3:** The comparison the phase composition of CuFe-1 and CuFe-2 according to the results of X-ray diffraction method (XRD) and differential dissolution (DD).

Sample	XRD (wt%)	DD (wt%)
CuFe-1	84% Cu_1−x_Fe_2+x_O_4_ (cub. spinel) 13% CuFeO_2_ 3% CuO	75.9% Cu_0.29_Fe_1_ ^1^ 14.4% Cu_1_ 9.7% Fe_1_
CuFe-2	73% spinel 6% Cu 12% CuO 9% Fe_2_O_3_	66.7% Cu_0.06_Fe_1_ ^2^ 29.3% Cu_1_ 4.0% Fe_1_

^1^ Corresponds to Cu_0.67_Fe_2.33_O_4_ formula. ^2^ Corresponds to Cu_0.16_Fe_2.84_O_4_ formula.

## Supplementary Materials

The following are available online at https://www.mdpi.com/article/10.3390/ma14185422/s1: Section: the preparation procedures of combustion products; Figure S1: the combustion synthesis of CuFe-1 and CuFe-5 samples as the process went on in time; Figure S2: the combustion synthesis of CuFe-2, CuFe-3 and CuFe-4 samples as the process went on in time; Figure S3: the combustion synthesis of CoFe sample as the process went on in time; Figure S4: typical kinetic curves of concentration of measured elements and changes of temperature and acidity of solvent on the time of sample dissolution in flow regime of DD experiment; Figure S5: kinetic curves of concentration of cobalt and iron and calculated Co/Fe molar ratio on the time of dissolution of CoFe sample; Figure S6: dynamics of dissolution of isolated phase of copper ferrite (Co_0.90_Fe_2.10_O_4_) and remaining phases of cobalt (oxide, metal) and iron (presumably Fe_2_O_3_); Figure S7: (a) kinetic curves of concentration of copper and iron and calculated Cu/Fe molar ratio, (b) dynamics of dissolution of isolated phase of copper ferrite (Cu_0.67_Fe_2.33_O_4_) and remaining phases of cupper (oxides, metal) and iron (presumably Fe_2_O_3_) during dissolution of CuFe-1 sample; Figure S8: (a) kinetic curves of concentration of copper and iron and calculated Cu/Fe molar ratio, (b) dynamics of dissolution of isolated phase of copper ferrite (Cu_0.16_Fe_2.84_O_4_) and remaining phases of copper (oxides, metal) and iron (presumably Fe_2_O_3_) during dissolution of CuFe-2 sample; Figure S9: SEM images for CuFe-1, CuFe-3 и CuFe-5 combustion products (Table 2).
