# Supplementary material for: Catalytic Behavior of Iron-Containing Cubic Spinel in the Hydrolysis and Hydrothermolysis of Ammonia Borane
Source: Materials (Basel). 2021 Sep 19;14(18):5422. doi: 10.3390/ma14185422 (PMC8468860; doi:10.3390/ma14185422)
Supplement: Supplementary file 1 [file materials-14-05422-s001.zip › materials-1376887-supplementary.pdf]

Supplementary Materials

Catalytic behavior of iron-containing cubic spinel in the hydrolysis and hydrothermolysis of ammonia borane

O.V.Komova, V.I.Simagina, A.A.Pochtar, O.A.Bulavchenko, A.V.Ishchenko, G.V.Odegova, A.M.Gorlova, A.M.Ozerova, I.L.Lipatnikova, E.S.Tayban, S.A.Mukha, O.V.Netskina

Boreskov Institute of Catalysis SB RAS, Pr. Akademika Lavrentieva 5, Novosibirsk 630090, Russia

The preparation procedures of combustion products

The following reagents were used: $Cu(NO_3)_2{\cdot}2.5H_2O$—98 wt.% (CAS 19004-19-4, Sigma-Aldrich); $Co(NO_3)_2{\cdot}6H_2O$—pure (GOST 4528-78, Ecros); $Fe(NO_3)_3{\cdot}9H_2O$—pure (GOST 4111-74, UzKhR); glycine (**Gly**) $C_2H_5O_2N$—pure (GOST 5860-75, Spektr-Khim); aqueous ammonia—special purity (GOST 24147-80, Baza №1 Khimreactivov); NH_4NO_3—analytically pure (GOST 22867–77, Promkhimkomplekt); HNO_3—special purity 18-4 (GOST 11125–84); $FeSO_4{\cdot}7H_2O$—analytically pure (GOST 4148-78, SoyuzKhimProm); $FeCl_3{\cdot}6H_2O$—pure (GOST 4147-7, SoyuzKhimProm); $CuCO_3{\cdot}Cu(OH)_2$—pure (GOST 8927-79, Reakhim); Co_3O_4—analytically pure (GOST 4467-79, SoyuzKhimProm); $NaBH_4$—purity of 98 wt% (CAS 16940-66-2, Chemical Line).

The CuFe-1 (S_{BET} = 4 $m^2{\cdot}g^{-1}$**)** sample was prepared by the self-propagating combustion of a dried and pelletized powder of the glycine-nitrate precursor in the air, as described in [1,2] (Figure S1). To prepare the precursor, an aqueous solution of ammonia (1.9 mL, ϱ = 0.908 g/mL) was added dropwise to 10 mL of an aqueous solution of $Cu(NO_3)_2{\cdot}2.5H_2O$ (1.165 g), $Fe(NO_3)_3{\cdot}9H_2O$ (4,04 g) and glycine (1.875 g) with the Gly : NO_3 : NH_3 molar ratio 1 : 1.6 : 1 with stirring. The resulting solution was slowly evaporated to the state of a gel and dried at 60 °C in a vacuum box. The CuFe-5 (S_{BET} < 1 $m^2{\cdot}g^{-1}$) sample was prepared by the same procedure without the addition of ammonia. For CuFe-1 and CuFe-5, the measured Cu/Fe molar ratios were 0.44 and 0.42, consequently.

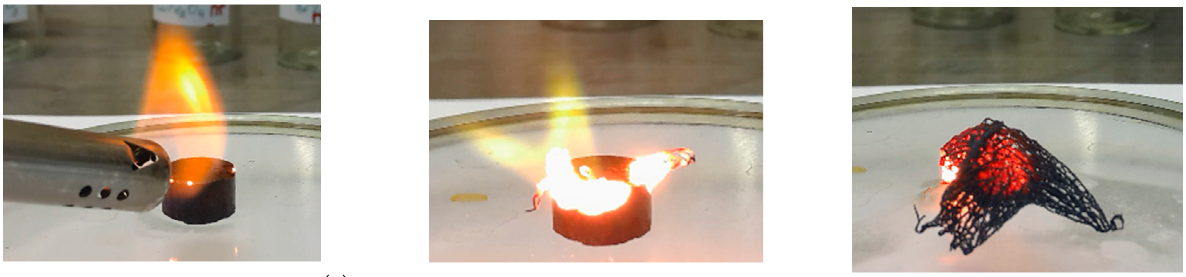

(a) (b) (c)

Figure S1. The combustion synthesis of CuFe-1 and CuFe-5 samples as the process went on in time.

To prepare the CuFe-2 sample (S_{BET} = 7 $m^2{\cdot}g^{-1}$, Cu/Fe = 0.44 in moles), 0.6978 g of $Cu(NO_3)_2{\cdot}2.5H_2O$, 2.424 g of $Fe(NO_3)_3{\cdot}9H_2O$ were combined with 1.134 g of Gly in a ceramic crucible (the crucible bottom diameter was 55 mm) and evaporated on a laboratory hot plate (C-MAG HS 4 digital (IKA, Staufen, Germany)) for 7 min at a preset temperature (T_{set}) of 100 °C. Then, 1.1 mL of an aqueous solution of NH_3 (ϱ = 0.908 g/mL) was added by drops to the brown homogeneous mass. The Gly : NO_3 : NH_3 molar ratio of the prepared precursor was 1 : 1.6 :1. The heating of the crucible with reactants was continued under stirring for 30 min at T_{set} = 150 °C which resulted in the evaporation of water to leave a thick homogeneous brown gel which hardened upon cooling down to ambient temperature. The layer-by-layer burning of the hardened gel was initiated at room temperature by its point-wise ignition with a glowing filament at the crucible wall (Figure S2). The combustion wave propagation from one crucible wall to the other took place during 24 s. The loose combustion product was subjected to an additional heating in air on the plate at T_{set} = 300 °C for 20 min.

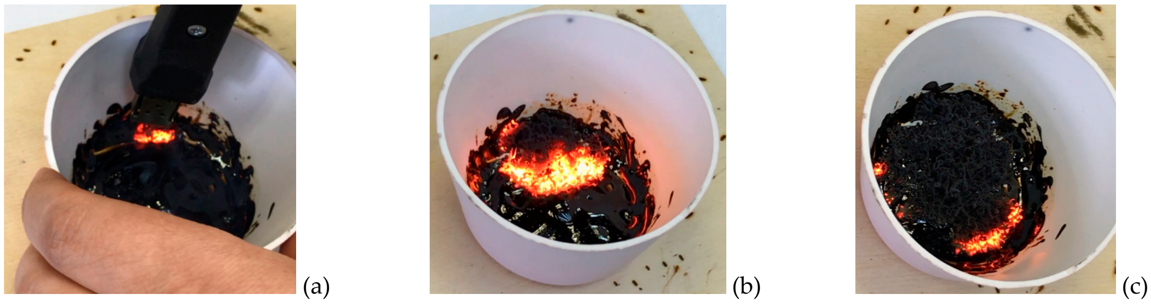

(a) (b) (c)

Figure S2. The combustion synthesis of CuFe-2, CuFe-3 and CuFe-4 samples as the process went on in time.

To prepare CuFe-3 sample (S_{BET}=7 $m^2 \cdot g^{-1}$, Cu/Fe = 0.43 in moles) with the molar ratio Gly : NO_3 = 1 : 2.6, 0.6978 g of $Cu(NO_3)_2 \cdot 2.5H_2O$, 2.424 g of $Fe(NO_3)_3 \cdot 9H_2O$ and 2.09 g of Gly·HNO_3 (for their preparation see below) were combined in a ceramic crucible and slowly evaporated at T_{set}=150 °C for 70 min under periodic stirring. The crucible was then allowed to cool down to room temperature and the layer-by-layer combustion of the hardened gel was performed as described above. The burning continued for 35 s. The formed product was also subjected to an additional heating in air at T_{set} = 300 °C.

To prepare the CuFe-4 sample (S_{BET} = 3 $m^2 \cdot g^{-1}$, Cu/Fe = 0.42 in moles), 0.6978 g of $Cu(NO_3)_2 \cdot 2.5H_2O$, 2.424 g of $Fe(NO_3)_3 \cdot 9H_2O$ and 2.35 g of Gly·NH_4NO_3 (for their preparation see below) were combined in a ceramic crucible and the slow process of evaporation was performed at T_{set} = 150 °C for 110 min under periodic stirring. The Gly : NO_3 : NH_4^+ molar ratio of the precursor was 1 : 2.6 :1. The process of combustion was as described above. With this precursor, the time of combustion was shortened to 9 seconds. Then, the product was additionally calcined at T_{set} = 300 °C.

As in the case of CuFe-1, for the synthesis of CoFe (S_{BET} = 4 $m^2 \cdot g^{-1}$, Cu/Fe = 0.44 in moles), a dry powder of the glycine-nitrate precursor was first prepared from water solution of $Co(NO_3)_2 \cdot 6H_2O$ (1.4552 g), $Fe(NO_3)_3 \cdot 9H_2O$ (4,04 g), glycine (1.89 g), aqueous ammonia (1.9 mL, ϱ = 0.908 g/mL). The Gly : NO_3 : NH_3 molar ratio of the precursor was 1 : 1.6 : 1. A dried powder of the precursor (1.7998 g) in a ceramic boat was allowed to stay in a heating box at 100 °C for 30 min which resulted in its melting to form a homogeneous layer. The hot product was taken out of the heating box and burned without allowing it to cool down (Figure S3). The combustion was started by igniting by means of a glowing filament. The front of combustion was moving along the entire length of the boat. The time of combustion was 38 s.

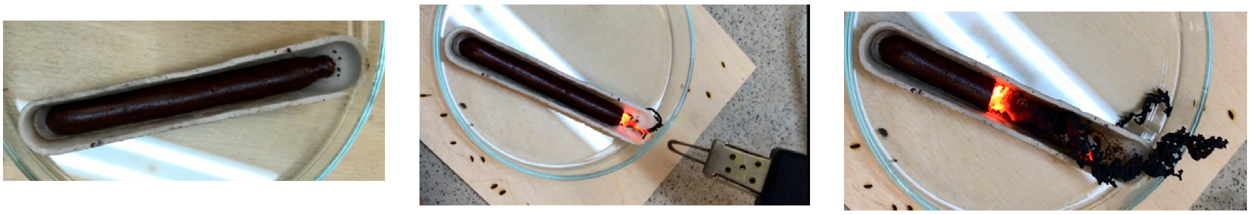

Figure S3. The combustion synthesis of CoFe sample as the process went on in time.

The samples designated as Gly·NH_4NO_3 and Gly·HNO_3 were synthesized from solutions of glycine and NH_4NO_3 (or HNO_3) taken in a 1 : 1 molar ratio. The resulting solution was slowly evaporated and dried at 60 °C in a vacuum box.

Differential dissolution (DD) experiment

The dissolution of cobalt ferrite (CoFe sample) was used as example

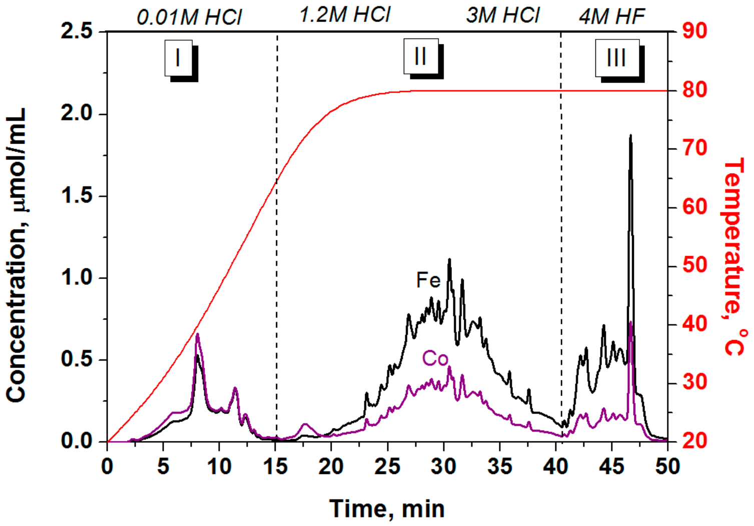

Figure S4. Typical kinetic curves of concentration of measured elements and changes of temperature and acidity of solvent on the time of sample dissolution in flow regime of DD experiment.

DD of the studied combustion products was carried out under a flow dynamic regime, i.e., under the conditions of a slow dissolution when the concentration, temperature and nature of the solvent were changed in the course of dissolution in order to gradually increase the dissolving power of the solvent (Figure S4). The solid phases of the studied material are successively dissolved in the solvent - from the easily dissolving phases to the poorly dissolving ones. The nature of the solvents must be such as to ensure complete dissolution of the product under study without the formation of precipitates and volatile compounds.

Methodology of isolation of cobalt ferrite phase at analysis of DD data

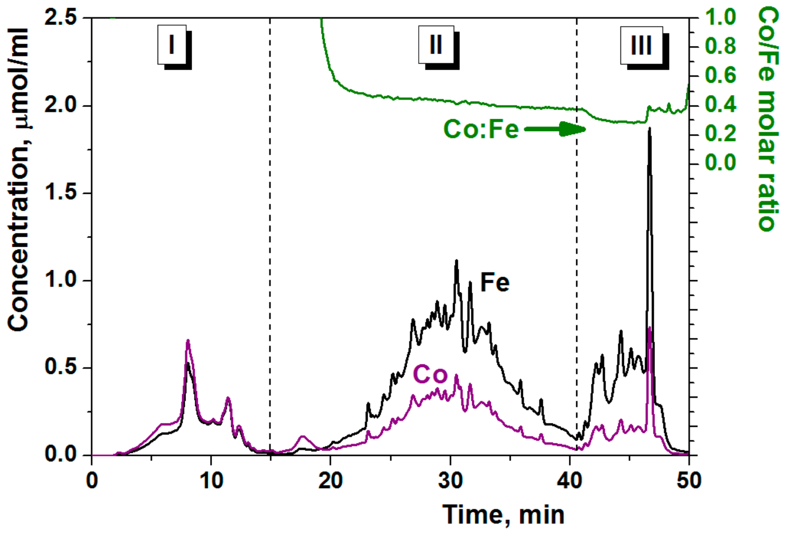

Figure S5. Kinetic curves of concentration of cobalt and iron and calculated Co/Fe molar ratio on the time of dissolution of CoFe sample.

The mathematical processing of the obtained kinetic curves was performed using specially developed domestic software. From the kinetic curves, the molar ratio Co/Fe was calculated to obtain a stoichiogram - the dependence of molar Co/Fe ration on the time of dissolution of the studied sample (Figure S5). At the start of the dissolution the Co/Fe profile tends to infinity which is an evidence of the presence on the surface of particles of the individual phases of cobalt oxide or metallic cobalt (Table 1).

The linear part on the Co/Fe stoichiogram (Co/Fe = 0.43 ± 0.02) indicates the formation of a mixed Co-Fe oxide phase. At the final stage of dissolution (after 40-50 min of the process) the molar ratio is Co/Fe < 0.43 which indicates simultaneous dissolution of the phase of the mixed oxide and the phase of the iron oxide.

Methodology of isolation of cobalt ferrite phase at analysis of DD data

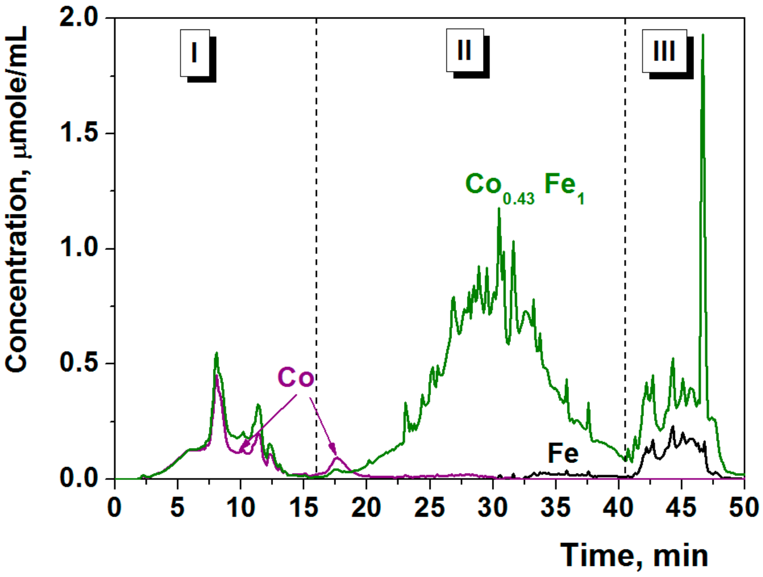

Figure S6. Dynamics of dissolution of isolated phase of copper ferrite ($Co_{0.90}Fe_{2.10}O_4$) and remaining phases of cobalt (oxide, metal) and iron (presumably Fe_2O_3).

After determination of the Co/Fe molar ratio in the cobalt ferrite phase (Figure S5) there was subtracting the main phase of the mixed Co-Fe oxide from the overall curves of dissolution of the measured elements (Co and Fe) and analyzing the dissolution of impurity phases (Figure S6).

As a result, the phase composition of CoFe can be given as follows:

$Co_{0.43}Fe_1$ – 87.4 wt%;

Fe_1 – 4.4 wt%;

Co_1 – 8.2 wt%.

Since this method is not able to determine the content of oxygen, the stoichiometric formulas of the phases are traditionally given without oxygen.

DD data for CuFe-1 sample

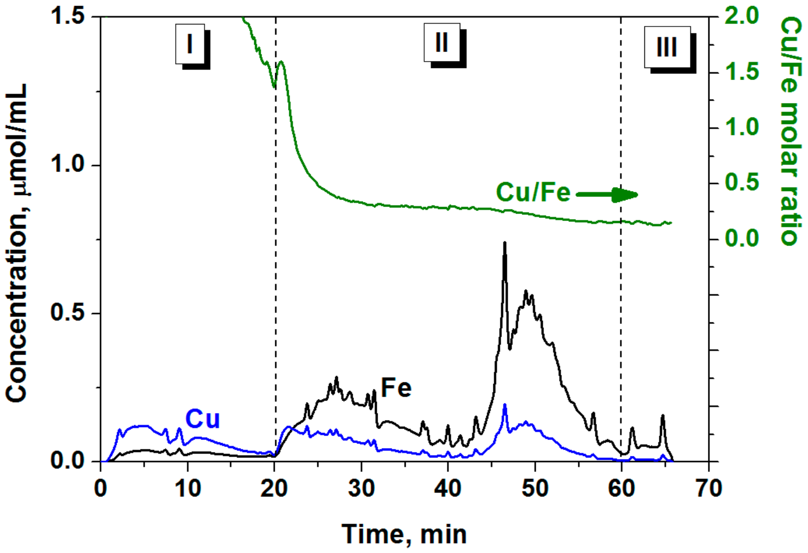

(a)

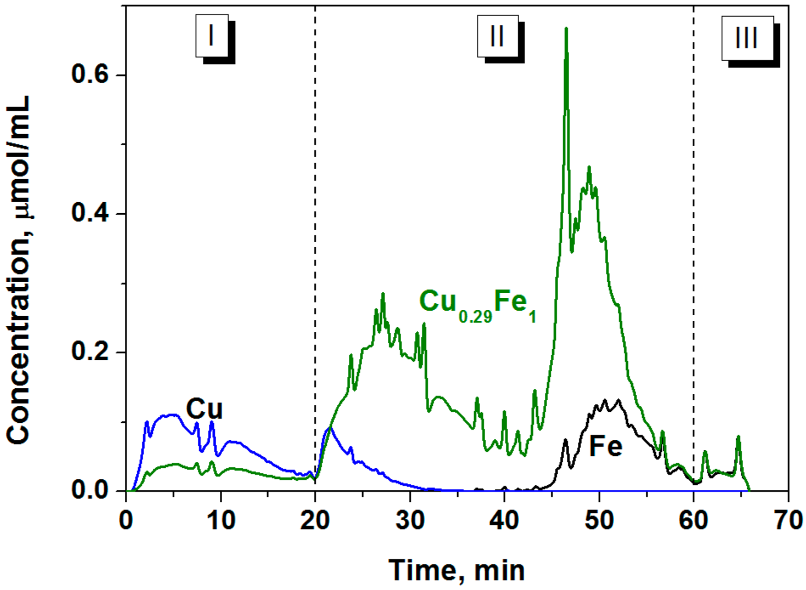

(b)

Figure S7. (a) Kinetic curves of concentration of copper and iron and calculated Cu/Fe molar ratio, **(b)** dynamics of dissolution of isolated phase of copper ferrite ($Cu_{0.67}Fe_{2.33}O_4$) and remaining phases of cupper (oxides, metal) and iron (presumably Fe_2O_3) during dissolution of CuFe-1 sample.

As a result (Figure S7), the phase composition of CuFe-1 can be given as follows:

$Cu_{0.29}Fe_1$ – 75.9 wt%;

Cu_1 – 14.4 wt%;

Fe_1 – 9.7 wt%.

DD data for CuFe-2 sample

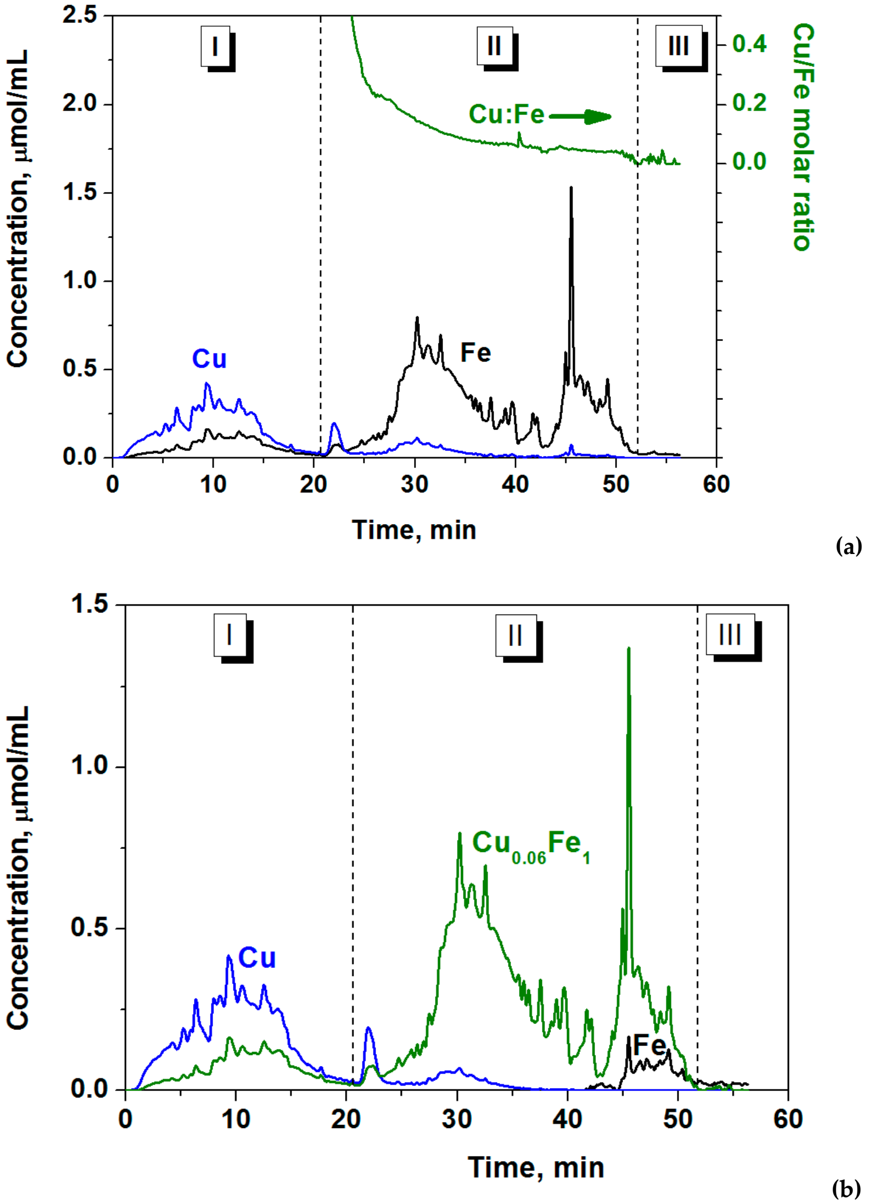

Figure S8. (a) Kinetic curves of concentration of copper and iron and calculated Cu/Fe molar ratio, **(b)** dynamics of dissolution of isolated phase of copper ferrite ($Cu_{0.16}Fe_{2.84}O_4$) and remaining phases of copper (oxides, metal) and iron (presumably Fe_2O_3) during dissolution of CuFe-2 sample.

As a result (Figure S8), the phase composition of CuFe-2 can be given as follows:

$Cu_{0.06}Fe_1$ – 66.7 wt%;

Cu_1 – 29.3 wt%;

Fe_1 – 4.0 wt%.

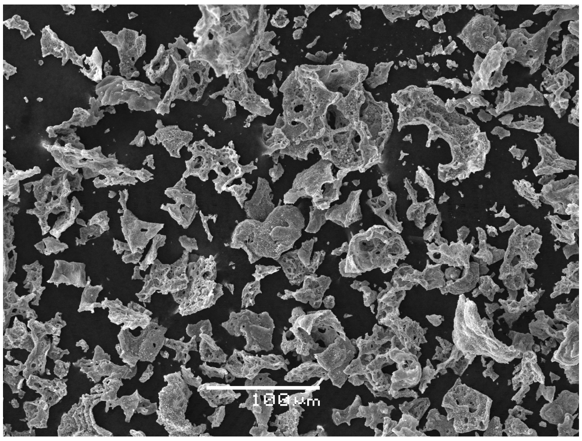

CuFe-1

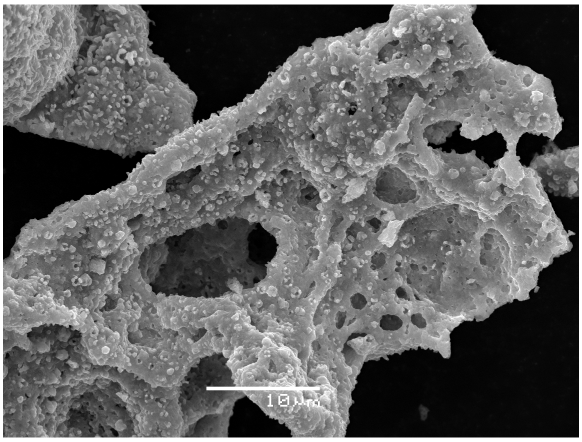

CuFe-1

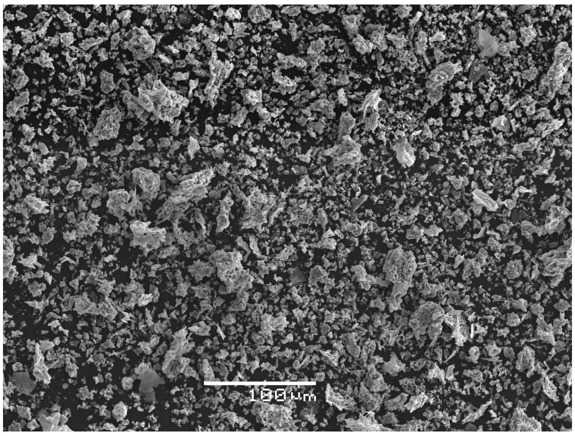

CuFe-3

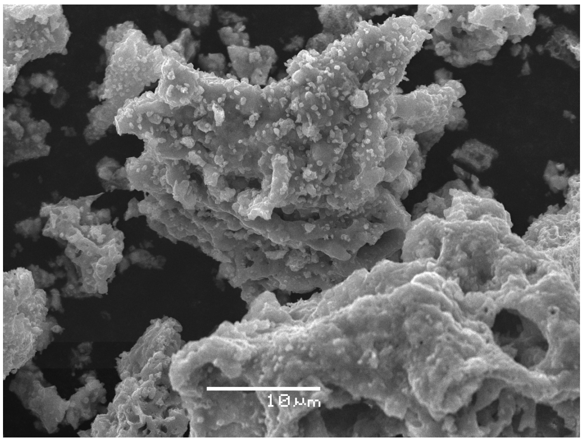

CuFe-3

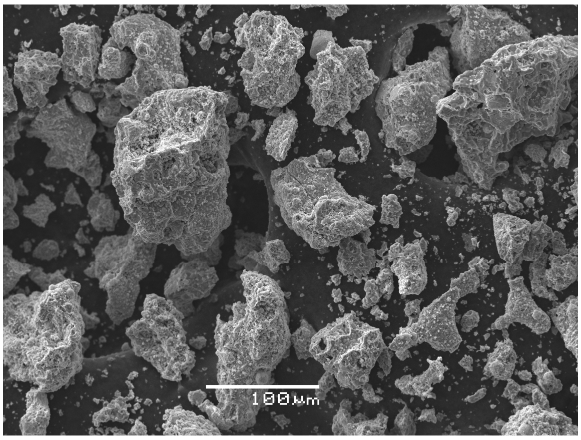

CuFe-5

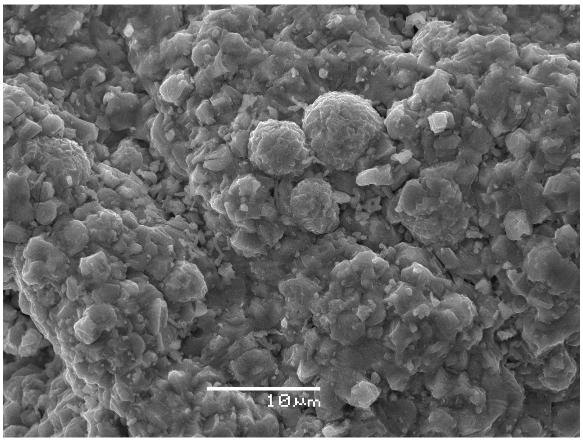

CuFe-5

Figure S9. SEM images for CuFe-1, CuFe-3 и CuFe-5 combustion products (Table 2).

References

1. Komova, O.V.; Odegova, G.V.; Gorlova, A.M.; Bulavchenko, O.A.; Pochtar, A.A.; Netskina, O.V.; Simagina, V.I. Copper–iron mixed oxide catalyst precursors prepared by glycine-nitrate combustion method for ammonia borane dehydrogenation processes. *Int. J. Hydrogen. Energy*. **2019**, *44*, 24277–24291, doi:10.1016/j.ijhydene.2019.07.137.
2. Simagina, V.I.; Komova, O.V; Odegova, G.V; Netskina, O.V Study of copper—iron mixed oxide with cubic spinel structure, synthesized by the combustion method. *Russ. J. Appl. Chem.* **2019**, *92*, 20–30, doi:10.1134/S1070427219010038.
